# Effect of ionic strength and seawater cations on hagfish slime formation

**DOI:** 10.1038/s41598-018-27975-0

**Published:** 2018-06-29

**Authors:** L. J. Böni, R. Zurflüh, M. E. Baumgartner, E. J. Windhab, P. Fischer, S. Kuster, P. A. Rühs

**Affiliations:** 1 Department of Health Science and Technology, ETH Zürich, 8092 Zürich, Switzerland; 2 Department of Materials, ETH Zürich, 8093 Zürich, Switzerland; 3 Department of Bioengineering and Materials Science and Engineering, University of California, Berkeley, California, 94720-1760 USA

## Abstract

The defensive slime of hagfish consists of a polyanionic mucin hydrogel that synergistically interacts with a fiber network forming a coherent and elastic hydrogel in high ionic strength seawater. In seawater, the slime deploys in less than a second entrapping large quantities of water by a well-timed thread skein unravelling and mucous gel swelling. This rapid and vast hydrogel formation is intriguing, as high ionic strength conditions generally counteract the swelling speed and ratio of polyelectrolyte hydrogels. In this work we investigate the effect of ionic strength and seawater cations on slime formation dynamics and functionality. In the absence of ionic strength skeins swell radially and unravel uncontrolled, probably causing tangling and creating a confined thread network that entraps limited water. At high ionic strength skeins unravel, but create a collapsed and dense fiber network. High ionic strength conditions therefore seem crucial for controlled skein unraveling, however not sufficient for water retention. Only the presence of naturally occurring Ca^2+^ or Mg^2+^-ions allowed for an expanded network and full water retention probably due to Ca^2+^-mediated vesicle rupture and cross-linking of the mucin. Our study demonstrates that hagfish slime deployment is a well-timed, ionic-strength, and divalent-cation dependent dynamic hydrogel formation process.

## Introduction

**Figure 1:**
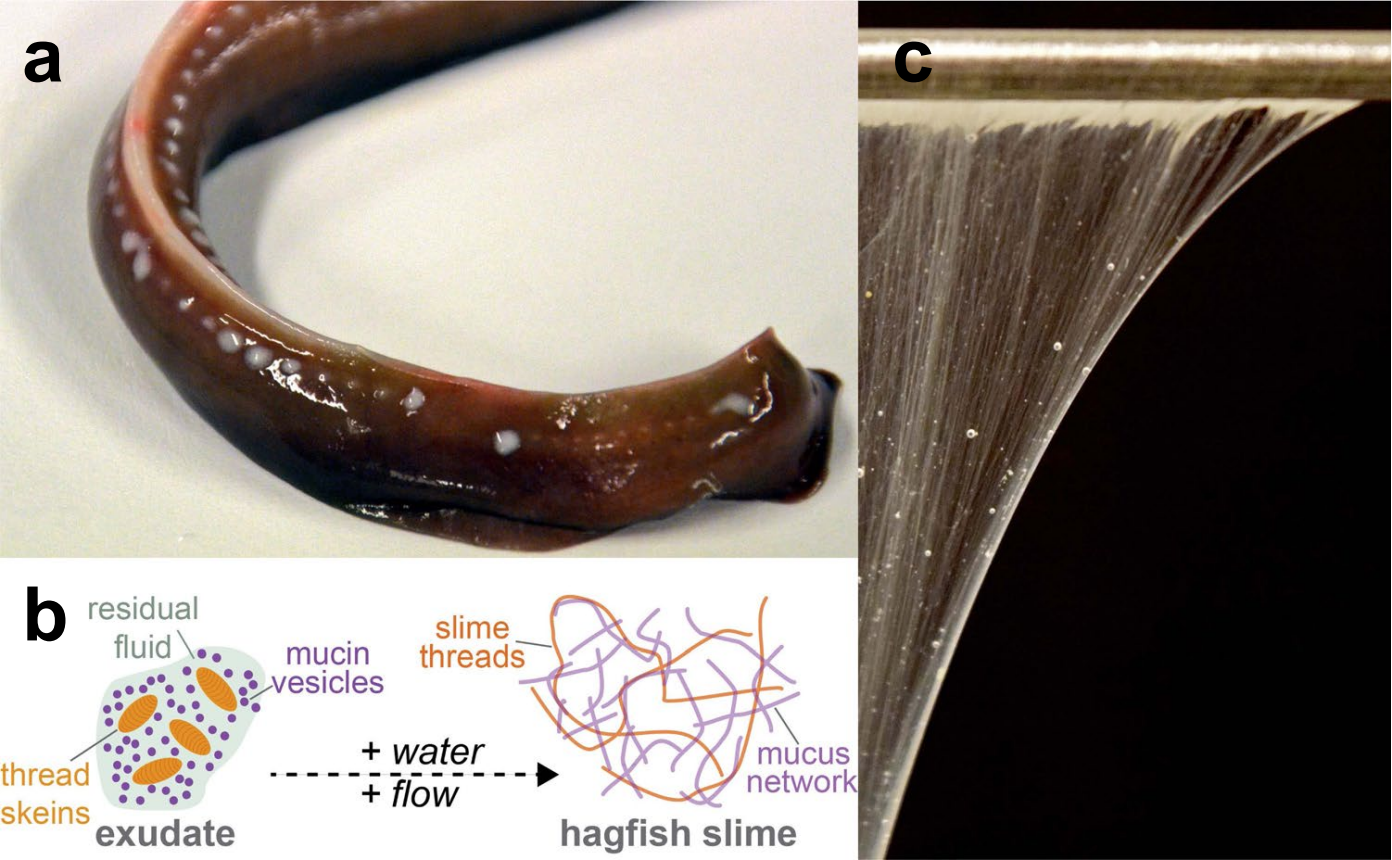
Overview of the components and the formation of hagfish slime. (**a**) Tail of an Atlantic hagfish (*M. glutinosa*). The ventro-lateral slime pores secrete white exudate from which the slime forms when in contact with seawater. (**b**) Schematic drawing of the slime formation mechanism. (**c**) Hagfish slime hanging on a spatula.

Hagfish are notorious for the vast amounts of slime they produce when provoked or attacked^1^. The slime serves as an immediate defense mechanism against potential predators^2^ by clogging their mouth and gills^3^. The slime forms when a glandular secretion, so-called exudate, is released into the surrounding seawater from a battery of ventrolateral pores (Fig. 1a). The whitish exudate consist of two major functional components: coiled-up threads (called skeins) and mucin vesicles (Fig. 1b). The thread skeins resemble a ‘ball-of-wool’ as they are made of a single coiled-up protein thread that is up to 30 cm long and 1–3 μm in diameter. When the skeins are ejected from the slime gland via holocrine secretion into the seawater they unravel and thus release their long intermediate filament bundle fiber, creating a fiber network^4,5^. The mucin vesicles contain negatively charged sulfonated mucin-like glycoproteins^6–8^. The vesicles swell, rupture, and release their mucus when exposed to water^9^. The hydrated mucus and the network of unraveled threads together form hagfish slime (Fig. 1c). The slime is an astonishing natural hydrogel as it is a highly elastic and coherent soft gel^10^ with a complex network structure that allows to physically confine up to 26’000 times its own weight in water^11^.

Hagfish slime constitutes a polyanionic mucin hydrogel that synergistically interacts with a fiber network in fragments of a second, rapidly forming a fibrous hydrogel in high ionic strength seawater (≈0.7 M^12^). The ability of a polyanionic gel to expand and swell vastly and rapidly in a high ionic strength solution is astonishing, because high salt levels generally counteract swelling of polyelectrolyte gels^13,14^. Polyelectrolyte gels can exhibit striking swelling degrees as high as 10^3^ in ion-free water relative to their dry weight, making them the preferred material for superabsorbents^15^. The swelling potential of these charged gels is defined by a balance between the elastic energy of the network counteracting swelling and the osmotic pressure of the ions as well as the electrostatic repulsion of the polymer-bound charges driving swelling^13,14,16^. The swelling capacity of polyelectrolyte gels is therefore much greater than that of neutral polymer gels because of the mutual electrostatic repulsion of polymer-bound charges as well as the osmotic pressure contributions of the counterions confined in the gel^17^. However, in the presence of salt electrostatic interactions in the gel are screened, causing a deswelling^13,18^. By contrast, hagfish slime forms in seawater in just a matter of seconds and entraps vast amounts of water.

Intrigued by the rapid and efficient swelling of hagfish slime in its natural high ionic strength environment, we investigate how hagfish slime formation is affected by the ionic strength and the cationic composition of water. The effect of ionic strength and ionic composition of the buffers used for hagfish exudate stabilization was studied before^8,19,20^ where it was found that high osmolarity buffers containing divalent anions (sulfate, citrate, phosphate) above a critical osmotic pressure of about 800 mOsmol l^−1^ stabilize hagfish exudate and thus prevent swelling of the gel. Other studies focused on the unraveling of skeins as a function of ionic strength, showing that skeins of the Pacific hagfish (*E. stoutii*) unraveled most at NaCl concentrations between 1–2 M^21^. Less proportions of bundles unraveled at lower (0.25, 0.5 M) and higher (2.5, 3, 4 M) NaCl solutions. In the same study the authors also found that seawater was superior to NaCl solutions in dissolving a glue that holds the skeins together and thus probably mediates unraveling once in water. In two other studies Herr *et al*.^9,22^ investigated the swelling and rupture of hagfish mucin vesicles in solutions containing different mono- and divalent salts and inorganic osmolytes. However, the effect of divalent salts and ionic strength on the formation and functionality of whole hagfish slime were so far only scantily investigated. Fudge^23^ showed that the removable mass of whole slime formed in distilled water and in 0.45 M NaCl solutions is substantially lower than in seawater. Also, the effect of NaCl solutions on skein unraveling was partly studied for *E. stoutii* skeins but the dynamic interactions of individual ion species during slime formation and especially their effect on the functionality of the resulting slime network are unknown.

In this work we combined material- and morphological characterizations to study the ionic strength dependent dynamics of hagfish slime. We show that ionic strength seems crucial for timing the slime formation via a controlled unraveling of the thread skeins. We further investigate the role of divalent seawater cations and show that their presence is vital in order to entrap large volumes of water in high ionic strength environments. In a last part we assess the flux of seawater cations (Na^+^, K^+^, Mg^2+^, Ca^2+^) during slime deployment, casting light on the complex cation dynamics during slime formation. Our insights might be valuable in the design of novel, bioinspired dynamic hydrogels that form rapidly in a high ionic strength environment with potential applications such as water desalination^24^.

## Materials and Methods

### Slime exudate sampling and stabilization

Atlantic hagfish (*M. glutinosa*) were fished by the staff of the Atlanterhavsparken in Ålesund, Norway (Supplementary Information Movie S1). Hagfish were captured in the Fjords of Ålesund in a depth of about 80 m, using custom built traps that were filled with fish scraps. The traps were lowered to the sandy bottom and remained there for about 2 h before they were reeled in. The captured hagfish were transferred to a seawater fed basin and subsequent slime exudate sampling was performed according to the approved ethical application by the Forsøksdyrutvalget (FOTS ID 6912) and followed a modified protocol of Herr *et al*.^9^ described by Böni *et al*.^25^. In brief, hagfish were anaesthetized using a 1:9 mixture of clove bud oil (Sigma, Switzerland) to ethanol, which was added to seawater in a 10 l bucket at a concentration of 1 ml of anesthetic per liter of seawater. The sedated hagfish were quickly transferred to a dissection tray, blotted dry, and slime exudate was obtained by mild electric stimulation (80 Hz, 18 V, HPG1, Velleman Instruments) on the ventral side. The released exudate was collected and stabilized in MCT oil (medium chain triglycerides, Delios GmbH, Germany) or in a high osmolarity citrate/PIPES (CP) buffer (0.9 M sodium citrate and 0.1 M PIPES at pH 6.7, 0.02% sodium azide and protease inhibitor, Sigmafast, Sigma) for mucin measurements and immediately stored at 4 °C. After sampling the fish were transferred to a recovery bath. Import of the samples was approved by the Swiss Federal Food Safety and Veterinary Office (FSVO) and export was approved by Norwegian Seafood Council. Viscosity measurements with hagfish mucins were done by mixing mucin vesicles suspension with seawater, diluted seawater, or Milli-Q. The vesicle suspension was prepared by filtering exudate in CP buffer through a series (60 and 20 μm) of nylon mesh filters (Merck, Germany) to separate the vesicles from the skeins. The vesicles were then concentrated by centrifugation at 3000 g for 10 min and the supernatant was discarded. The mucin content of the vesicle suspension was determined in triplicates by dialysis (25 kDa MWCO, SpectraPor, USA), dialyzing 0.5 ml of the vesicle solution against Milli-Q (three batches, 12 h each) and subsequent freeze drying to obtain the dry weight^8^. The mucin concentration of the stock suspension was 2.6 ± 0.8 mg ml^−1^.

### Water retention

Water retention measurements to assess slime functionality were performed according to a protocol of Böni *et al*.^25^. In brief, slime was prepared by placing 4 μl of MCT stabilized exudate on the bottom of a 20 ml glass flask with a micropipette. Subsequently, 20 ml liquid were poured in, the lid was closed, and the flask was gently turned upside-down eight times. The deployed slime was transferred from the glass flask to a small beaker and placed on a laboratory scale, which had an in-house built mixing device attached on top and a video camera (Sony alpha 5100) placed in front to optically monitor the weight changes. The mixing device was then slowly lowered into the slime and rotated ten times to wrap up the slime mass. The wrapped slime was lifted up, the device arrested in the upper position, and the water egress recorded gravimetrically for five minutes. All experiments were performed in triplicates. The exudate concentration in all measurements was 0.02 wt% and was determined assuming a density of 1 g ml^−1^ exudate^10^. Seawater for all experiments was obtained in the Fjords of Ålesund and was sterile filtered (0.45 μm, cellulose acetate sterile syringe filter, VWR, USA) prior to measurements. Artificial seawater (ASW) was prepared according to a recipe of Kester *et al*.^26^, shown in the Supplementary Information (Table S1). All salts were obtained from Sigma. The ionic strength I of all solutions (Supplementary Information Table S2) was calculated using the equation $I=\frac{1}{2}{\sum }_{1}^{n}{c}_{i}{z}_{i}^{2}$, where c is the concentration of the dissolved salt ion in mol l^−1^ and z is the valency of the ion. For the dissolved salts a complete dissociation was assumed.

### UV-VIS

Turbidity scales with the amount of condensed vesicles in a solution and can be used to monitor their presence as found by Salo *et al*.^8^. Absorption measurements were performed at 350 nm using a UV-VIS spectrophotometer (Cary 300, Agilent Technologies, USA). NaCl solutions of different molarities were poured over fresh, MCT-oil stabilized exudate without mixing. Because no mixing was applied many skeins did not open, especially at higher NaCl concentrations. The solutions were let to rest for 1 hour, allowing for unopened skeins to sediment. The condensed vesicles will not sediment within this time frame because of their smaller size. 1 ml liquid was then taken from the top and filled into cuvettes for turbidity measurement. After a first measurement, 10 μl of a 1 M CaCl_2_ solution was added to the cuvette to reach a final Ca^2+^ concentration of 10 mM. The liquid in the cuvette was gently mixed and the turbidity measured again. All measurements were performed in triplicate, at room temperature, and at an exudate concentration of 0.8 μl exudate ml^−1^.

### Microscopy

Light microscopy was performed on a Nikon Diaphot (Nikon, Japan) in transmission illumination mode using 20× and 40× magnification objectives. Images and videos were captured and analyzed with the NIS elements D3.0 software. High speed image sequences of skein unraveling were captured with a high speed camera (Memrecam fx RX6, nac Image Technology, USA) connected to the light microscope. Movies were recorded at a framerate of 1000 frames per second and a shutter speed of 20 kHz.

### Dynamic viscosity measurements

For dynamic viscosity measurements the mucin solution was analysed using a capillary viscometer (KPG Ubbelohde, SI Analytics GmBH, Mainz, Germany) with a capillary Nr. II with a diameter of 1.13 mm. Capillary viscometers work on the basis of the Hagen-Poiseuille law $\frac{V}{t}=\frac{\pi {R}^{4}\Delta P}{8L\mu }$ in which t, V, R, L, ΔP, and μ are time, volume, radius of capillary, length of capillary, pressure drop, and dynamic viscosity of fluids, respectively. The viscosity is determined by measuring the time required for a defined liquid volume to flow through a capillary tube. The sample was loaded and drawn up through the capillary by applying a vacuum with a syringe. Subsequently, the vacuum was released and the time needed for the mucin solution to pass the graduation marks was measured with a precision stopwatch. By reordering the Hagen-Poiseuille law, the kinematic viscosity v can be calculated as ν = K (∆t − Θ) where K is the constant for the capillary (in this case K = 0.1 mm^2^ s^−2^), ∆t the time used for the passage, and Θ is the Hagenbach correction (HC) time, which depends on the capillary diameter and the passage time and is provided by the supplier. The dynamic viscosity was then calculated as η = v ρ with ρ being the fluid density. Mucin concentration was about 0.02 mg ml^−1^. All samples were measured in triplicates and at room temperature.

### Atomic absorption spectroscopy (AAS)

The concentration of cations (Na^+^, K^+^, Ca^2+^, Mg^2+^) in seawater and in seawater that interacted with hagfish slime was determined using flame atomic absorption spectroscopy (AAS). The cation concentrations were determined for seawater, seawater that remained in the glass after gelation and immediate removal of the slime (‘unbound fraction’), and for seawater that drained from hagfish slime during five minutes (‘bound and drained fraction’). Slime samples were prepared as for the water retention measurements and slime was removed using the in-house built mixing device described above. Flame AAS was performed on a Varian AA240FS Fast Sequential Atomic Absorption Spectrometer (Agilent Technologies AG, Switzerland). The hollow cathode lamp was heated for 30 minutes before measuring. For calibration, 3 different commercial standards (Merck; Titrisol) and a zero were freshly prepared with Milli-Q water and 100 μl of 32% hydrochloric acid (HCl). The stock solutions of the standards were diluted to 0.25–5 ppm, depending on the cation and the detection range of the individual lamp. The sampling tube was flushed with Milli-Q water between all measurements. All AAS data was measured within the confidence interval and the calibration range of the respective cation. As performed with the calibration standards, all samples were acidified with 32% HCl prior to dilution for the measurement to ensure full dissolution of the contained metals. A repeated measures ANOVA was performed with SPSS. The data fulfilled all statistical assumptions. All experiments were performed in triplicates.

### Data availability

All data generated or analysed in this study are included in this published article and its Supplementary Information files.

## Results and Discussion

**Figure 2:**
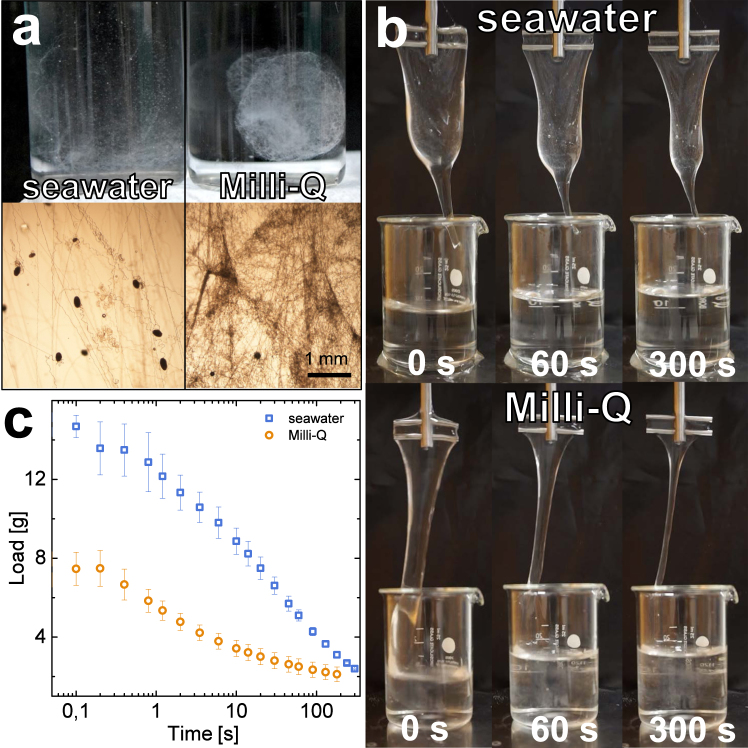
Effect of seawater and Milli-Q on hagfish slime thread network and water retention properties. (**a**) Hagfish slime formed with seawater (left) and Milli-Q (right). In seawater the expanded thread network spans the entire volume whereas in Milli-Q a confined ‘blob’-like network is formed. (**b**) Images of slime draining during water retention measurements. (the image series in the top line in (**b**) was previously published^25^). (**c**) Water retention measurements of slime formed in seawater and Milli-Q.

The effect of ionic strength on hagfish slime was evaluated by mixing freshly harvested hagfish exudate into Milli-Q water and seawater, representing the complete absence of ions and the natural environment for hagfish slime formation, respectively. The resulting fiber network was investigated under the microscope and the water retention of the slime was measured. Milli-Q water resulted in the formation of a confined and compact slime mass, showing a close arrangement and narrow spacings between the slime threads instead of a widespread and expanded network as observed in seawater (Fig. 2a). The slime formed in Milli-Q did not span the entire available water volume of 20 ml upon deployment in the glass flask, which resulted in a condensed ‘blob’. Furthermore, almost all skeins unraveled in Milli-Q, which stands in contrast to observations in seawater where skeins have been shown to remain coiled^27^. Although more skeins unraveled in Milli-Q, the slime showed a substantially reduced ability to entrap water (Fig. 2b). Slime in Milli-Q initially retained 7.5 g of water, which is roughly 50% less compared to the 14.7 g retained in seawater (Fig. 2c). We suggest that this effect is caused by altered network formation dynamics. In the following sections, we will discuss the effects of ionic strength and seawater cations on skein unraveling, vesicle decondensation, and mucin viscosity and will suggest their implications on whole slime formation and functionality.

### Unravelling dynamics of thread skeins in Milli-Q and seawater

**Figure 3:**
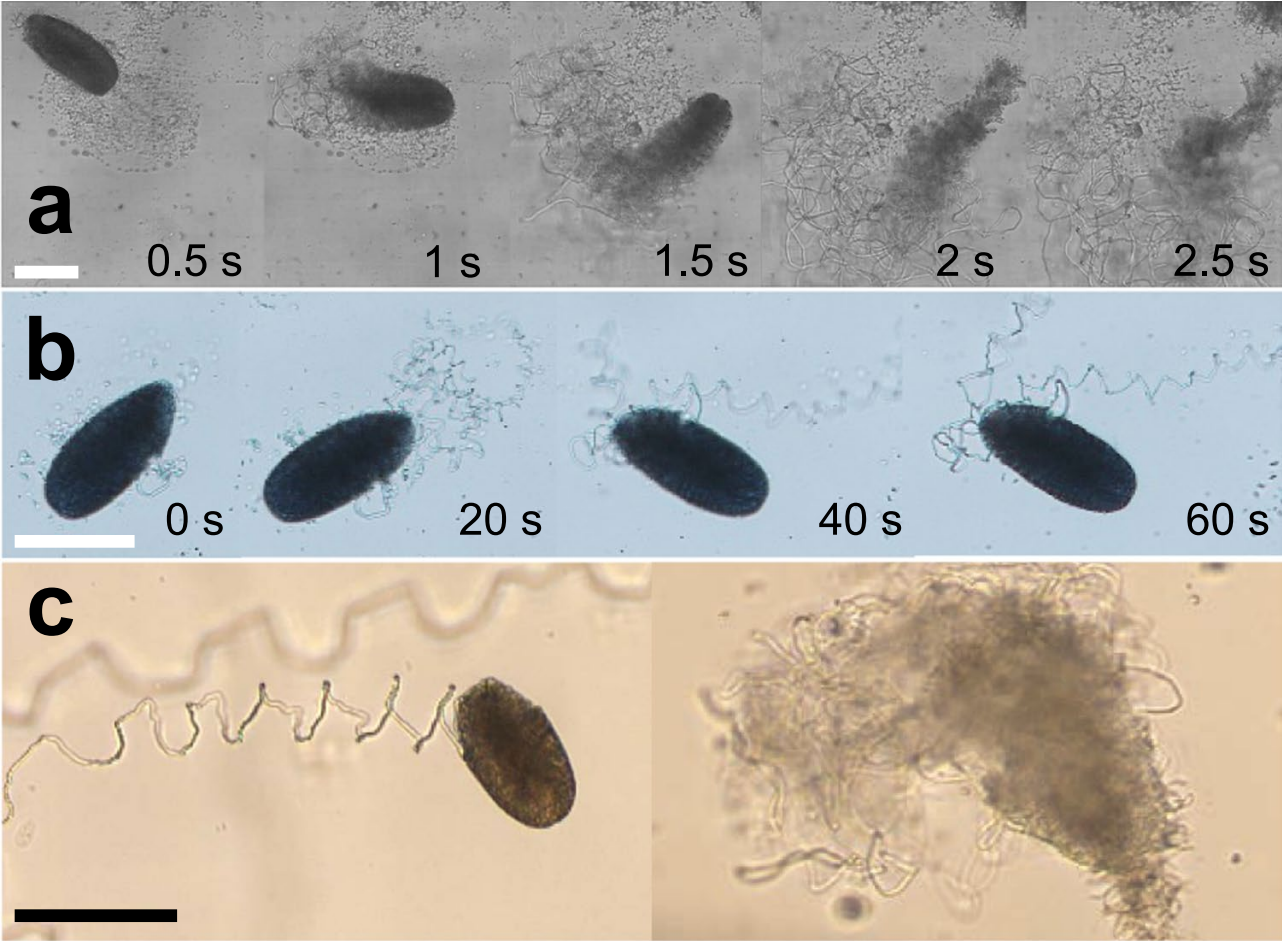
Characteristic unraveling times of single *M. glutinosa* skeins in ion-free Milli-Q water and in seawater observed under a light microscope. (**a**) Image series of a skein unraveling in Milli-Q captured with a high speed camera. (**b**) Skein unraveling in seawater. (**c**) Comparison of skeins unraveling with seawater (left) and Milli-Q (right), showing the radial swelling in Milli-Q and the controlled unraveling from the apical tip in seawater. Scale bars = 100 μm.

Skein unraveling dynamics in ion-free Milli-Q are less controlled and faster than in seawater. Figure 3a shows an image sequence of a skein unraveling in Milli-Q (Supplementary Information Movie S2). The skein swells radially and completes unravelling in about two seconds, even in the absence of substantial external flows. Similar to seawater, the unraveling started at the apical tip of the skein^21,25^, but then the skein continued to uncoil from both sides in an uncontrolled manner. As the uncoiling is localized to the position of the skein, a confined and narrow thread mesh remains on the uncoiling spot. In contrast, unraveling in seawater can take several minutes when observed under the microscope^21,25^ (Fig. 3b) and the uncoiling threads are able to span a larger area, especially in the presence of flows. A direct comparison of the skein unraveling characteristics in seawater and in Milli-Q is shown in Fig. 3c. The slower unraveling of skeins of the Atlantic hagfish (*M. glutinosa*) compared to skeins of the Pacific hagfish (*E. stoutii*) in seawater was similarly observed by Bernards *et al*.^21^, who suggested that a slime that deploys too fast could have drawbacks for burrowing animals such as the Atlantic hagfish.

In Milli-Q the combination of radial swelling and uncontrolled unraveling from two sides probably causes the uncoiling skeins to tangle. Tangled threads are limited in their ability to spread out after they have unraveled, which in turn impairs the formation of a widespread and expanded network, resulting in a lower water retention.

### Ionic strength determines skein unraveling

Seawater and Milli-Q resulted in distinct network properties and skein unraveling. We studied the effect of ionic strength on skein unraveling and found that ionic strength slows down unraveling speed and reduces swelling of the skeins. Radial swelling and uncontrolled, fast unraveling of skeins was observed in low-ionic strength sodium chloride solutions (10 mM NaCl, I = 0.01 M) and in diluted seawater (10% seawater, I = 0.06 M), similar to Milli-Q (Supplementary Information Movie S3). The presence of increased ionic strength in the form of sodium chloride (100 mM NaCl, 500 mM NaCl), seawater, or artificial seawater lacking certain cations in contrast resulted in controlled (only from the apical end) and slow unraveling of the skeins (Supplementary Information Movie S4). We have two hypotheses how a high ionic strength could slow down and control skein unraveling. The two hypotheses are not mutually exclusive and both give a reasoning to slower unraveling in a high ionic strength environment.

First, the fast unraveling and radial swelling of the skeins in ion-free water could be caused by thread swelling given the large osmotic gradient. Salt dependence in thread skein uncoiling between distilled water and seawater was already observed and a dependence of uncoiling on seawater-induced swelling of thread skeins was suggested^4,28^. Milli-Q could cause an excessive swelling of the keratin-like^29–31^ hagfish slime threads. Unlike in hard α-keratins, where intermediate filaments (IFs) are embedded in a isotropic, high-sulfur matrix^32^, hagfish slime threads constitute matrix-free IFs and are therefore highly sensitive to hydration^33,34^. Fudge and Gosline^34^ showed that hagfish threads increased 45% in diameter compared to the dry state when hydrated with deionized water. As spontaneous unraveling of the skeins is considered to be propelled by a stored strain energy in the coiled thread, it seems probable that an excessive swelling of the thread adds to this stored entropy-elasticity. The osmolarity of seawater - as well as the high osmolarity of the residual fluid (888 mOsm) of the slime exudate^9^ - could reduce the swelling of the threads compared to Milli-Q and thus limit the strain energy, resulting in a slower and controlled unraveling, which can then be accelerated by external mixing flows and attaching mucus strands.

The second hypothesis is that ionic strength reduces the dissolution speed of the seawater-soluble glue, which was found to mediate unraveling in *E. stoutii* skeins^21^ and similarly observed on *M. glutinosa* skeins^25^. It is possible that the glue dissolves faster in the presence of low ionic strength solutions and that its dissolution speed is reduced at high ionic strength. Deionized water seems to be sufficient to loosen the glue from binding to itself, as already shown in electron microscopy images^25^. However, a low ionic strength could prevent a further dissolution of the glue from the threads as observed by Bernards *et al*.^21^. Both hypotheses could also explain why skeins are stable in high-osmolarity stabilization buffers^8,19,20^; given a suggested increased insolubility of the glue and/or an osmotically dehydrated slime thread with reduced strain energy.

Regardless if one or both suggested hypotheses are considered the main reason for the observed differences in skein unraveling, the different unraveling patterns governed by ionic strength have implications on whole slime functionality.

### Effects of ionic strength on slime network formation

**Figure 4:**
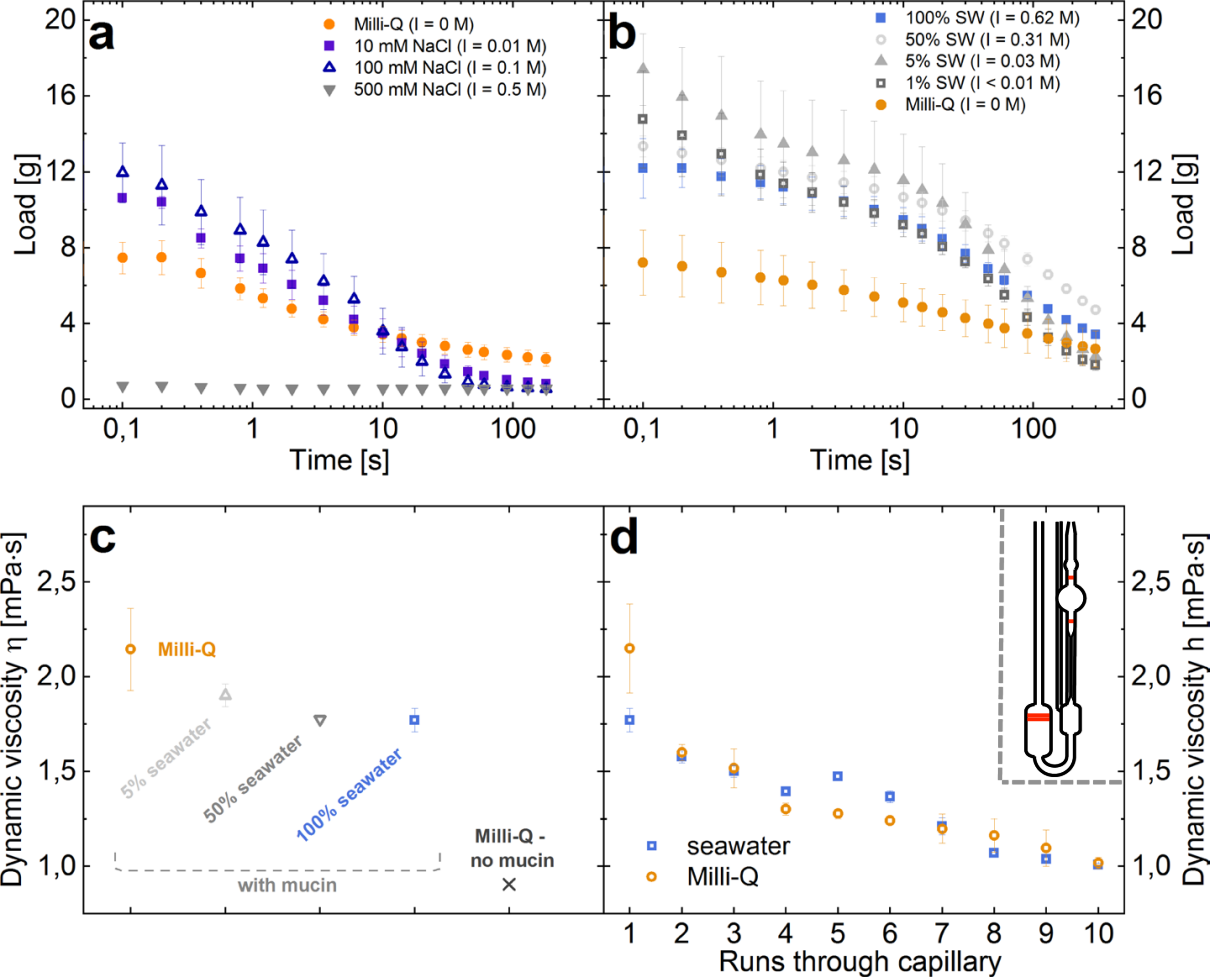
Effect of ionic strength on the water retention properties of hagfish slime and on mucin viscosity. (**a**) Water retention in different concentrations of sodium chloride (NaCl). (**b**) Water retention of hagfish slime formed in dilutions of seawater. For comparison between NaCl and seawater measurements, the ionic strength I is indicated in brackets. (**c**) Dynamic viscosity of hagfish mucin in Milli-Q, seawater, and diluted seawater at a concentration of 0.02 mg/ml measured at room temperature. Pure Milli-Q is given as a reference. (**d**) Mucin viscosity as a function of runs through the capillary of the viscometer, showing the mechanical sensitivity of hagfish mucin towards shear. The inlet shows a schematic drawing of an Ubbelohde capillary viscometer.

Similar to skein unraveling, the effect of ionic strength on whole slime functionality was studied by mixing hagfish slime exudate with solutions of various sodium chloride (NaCl) concentrations and dilutions of natural seawater. The presence of 10 to 100 mM NaCl resulted in a substantially increased initial load compared to Milli-Q (Fig. 4a) and the fiber network did not show a clump formation after mixing, supporting the beneficial effect of ionic strength. The observed differences between the treatments were too large to be accounted for simply by differences in the density of the different salt solutions. Although a low ionic strength (10 mM NaCl and up to 10% seawater) showed skein swelling under the microscope, the salts had beneficial effects on the water retention. However, a high ionic strength solely based on sodium chloride (I = 0.5 M) - being close to the ionic strength of natural seawater (I = 0.6–0.7 M) - resulted in a collapsed and dysfunctional slime. Although single skeins did not swell and unraveled controlled under the microscope (Supplementary Information Movie S4), no widespread fiber network formed (Supplementary Information Figure S1a) and no water was retained.

This stands in contrast to seawater, where the slime shows a functional network and superior water retention properties despite a high ionic strength (Fig. 4b). Even in the presence of 1% seawater (I < 0.01 M) the initial load was increased to ≈12 g compared to ≈7 g in Milli-Q and ≈10 g in 10 mM NaCl (Fig. 4b). 5% seawater retained the most water initially over 100% seawater. These findings imply an important role of other seawater cations such as Ca^2+^ and Mg^2+^ for slime functionality in a high ionic strength environment, which will be discussed in the following section. Furthermore, it suggests that slime network functionality is not determined by skein unraveling and ionic strength alone and that the dynamics of vesicle rupture and mucin viscosity might be similarly crucial.

Mucin viscosity measurements (Fig. 4c) showed that in Milli-Q hagfish mucin had the highest viscosity (2.14 mPas). At increased ionic strength as the case in 5% and 100% seawater the viscosities dropped to 1.86 mPas and 1.77 mPas, respectively. Theses results are in good agreement with the findings of Fudge *et al*.^11^ who measured a viscosity of 1.41 mPas in seawater and about 1.54 mPas in Milli-Q at 9 °C. The higher viscosity of mucin in 5% seawater compared to 100% seawater could explain why 5% seawater showed a higher initial load over 100% seawater in water retention measurements (Fig. 4b). A higher viscosity means a higher resistance to flow, suggesting that liquid should be better retained. A higher viscosity combined with the presence of small amounts of salts in 5% seawater and their beneficial effect on skein unraveling seem to lead to a slime with superior water retention properties in comparison to slime formed under natural conditions. However, this does not infer that slime formed in 5% seawater also has superior defense properties. Although Milli-Q showed a higher viscosity than all dilutions of seawater, water retention in Milli-Q was inferior to seawater (Fig. 2c). In this case the negative effects of the proposed tangling of the uncoiling skeins on network formation probably outbalances the slightly positive effect of viscosity on water retention.

The lower mucin viscosity in seawater compared to Milli-Q probably originates in increased electrostatic charge suppression^35,36^ of the high ionic strength in seawater. Similar effects were shown for porcine gastric mucin (PGM), which does not gel at high ionic strengths (>0.1 M)^36^ and for human sputum, which shows reduced spinnability, rigidity, and viscoelasticity after treatment with hypertonic saline solutions^37–40^. Polyelectrolyte gels such as mucins are shown to stiffen as they swell in low salt solutions because the counterions in the gel network increase the internal pressure^41^, thus increasing in viscosity. Furthermore, hagfish mucin viscosity showed a sensitivity towards mechanical shear (Fig. 4d), regardless whether in seawater or in Milli-Q. The sensitivity of hagfish slime towards mechanical stress is well known^3,42^ and was similarly shown for hagfish mucin using a rotational shear rheometer^43^. These results support previous observations that hagfish mucin seems to aggregate under shear^43^ and suggest that network cross-links could be disrupted. For the capillary rheometry experiments the mucin solution had to be pulled up through the glass capillary in order to prepare the measurement, meaning the mucin solution inevitably already experienced one shear event prior to the measurement. This infers that the viscosity of natural hagfish mucin immediately after secretion could be substantially higher than reported so far.

### Divalent seawater cations (Ca^2+^ and Mg^2+^) are crucial for whole slime functionality

**Figure 5:**
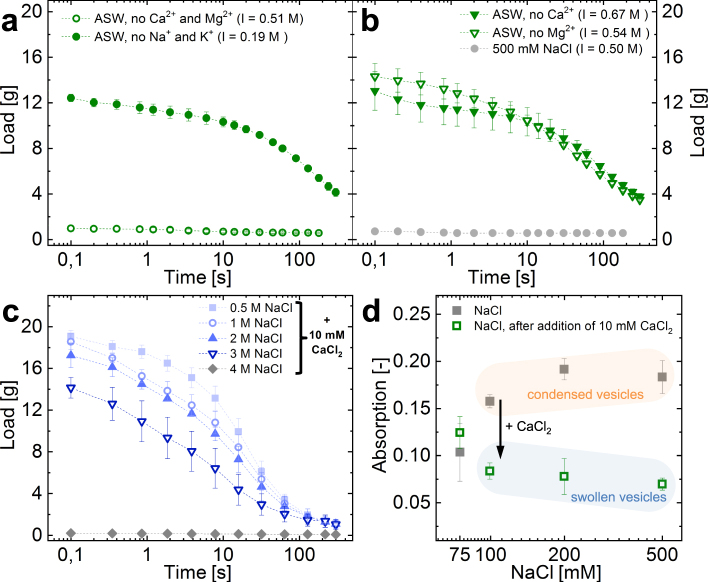
Effect of ionic composition of various versions of artificial seawater (ASW) on slime functionality. (**a**) Water retention in ASW without divalent cations (Ca^2+^ and Mg^2+^) and ASW without monovalent cations (Na^+^ and K^+^). (**b**) Water retention in ASW either without Ca^2+^ or Mg^2+^, showing that one divalent ion is sufficient for functionality at a high ionic strength. If none are present at a comparable ionic strength, no water is retained (500 mM NaCl). For comparison the ionic strength (I) for every measurement is provided in brackets. (**c**) Water retention in NaCl solutions containing 10 mM CaCl_2_. (**d**) UV-VIS turbidity measurements of hagfish exudate mixed with NaCl solutions in the absence of calcium. The turbidity at NaCl ≥100 mM originates in the presence of condensed vesicles and vanished once 10 mM CaCl_2_ are added.

The importance of divalent seawater cations (Ca^2+^ or Mg^2+^) to efficiently entrap water in a high ionic strength environment was investigated using artificial seawater (ASW) and modifications thereof, lacking specific cations. Water retentions of hagfish slime formed with natural seawater and with ASW did not show substantial differences (Supplementary Information Figure S2a), despite the differences in cationic composition (Supplementary Information Table S3). In contrast, ASW lacking divalent cations Ca^2+^ and Mg^2+^ did not form a functional slime network (Fig. 5a, Supplementary Information Figure S1b), i.e. no water was entrapped as similarly observed for the 500 mM NaCl solution (Fig. 4a). Also, when EDTA - being a strong chelator of di- and trivalent cations - was mixed into seawater, the initial entrapped load dropped significantly (Supplementary Information Figure S2b). In contrast, ASW lacking monovalent seawater cations (Na^+^ and K^+^) but containing the divalent seawater cations (Ca^2+^ and Mg^2+^) resulted in a slime that efficiently entrapped and retained the water, similar to seawater. We found that when one of the two major divalent cations was present at its natural concentration like in seawater (10 mM Ca^2+^; 50 mM Mg^2+ ^^26^), a functional slime network was formed, which entrapped and retained water (Fig. 5b). The beneficial effect of calcium ions was found to allow slime formation beyond ionic strengths occurring in natural seawater. In the presence of 10 mM Ca^2+^ functional slime networks formed in solutions containing up to 3 M NaCl (Fig. 5c), corresponding to about 4–5 times the ionic strength occurring in natural seawater. However, slime formation eventually failed at 4 M NaCl. Similarly, Bernards *et al*.^21^ showed that skein unraveling is inhibited in 4 M NaCl in Pacific hagfish slime. The initial load slightly decreased with increasing NaCl molarity, which could originate in a lower mucin viscosity due to charge screening and/or in the higher density of the higher molarity fluids. These measurements show the extreme resilience and the limits of hagfish slime to high salt conditions and underline the importance of calcium.

The crucial role of the divalent cation Ca^2+^ for mucin vesicle rupture was in depth investigated by Herr *et al*.^22^. The authors showed that Ca^2+^ is required for the swelling and rupture in approximately 60% of vesicles in seawater strength osmolarity and suggested that Ca^2+^-activated transporters in the vesicle membrane are responsible for the need of calcium. The remaining 40% ruptured also in the absence of Ca^2+^. All vesicles ruptured when exposed to distilled water^9^. Our observations are in line the findings of Herr *et al*.^22^ and show that calcium is needed for complete vesicle decondensation already at NaCl concentrations approximately ≥100 mM (Fig. 5d). The turbidity at 75 mM NaCl did not significantly change upon calcium addition, suggesting that most vesicles swelled even in the absence of calcium. At 50 mM the solution was already viscous and many skeins unraveled, making turbidity measurements difficult (not shown). However, the onset of viscosity and unraveled skeins suggest that in these conditions most vesicles swelled and ruptured.

These findings imply that a low ionic strength (approx. ≤100 mM) allows a hypo-osmotic swelling and rupture of most mucin vesicles similar to Milli-Q but results in a controlled skein unraveling as the ionic strength could be sufficient to suppress excessive thread swelling. Combined, this seems to form a somewhat functional fiber network that retains more water than Milli-Q (Fig. 5b). At a high ionic strength (approx. ≥100 mM) vesicle decondensation is limited to about 40% of the vesicles. The reduced amount of ruptured vesicles and mucin strands does not seem to be able to sufficiently drive the unraveling of the skeins. A strongly impaired and collapsed network forms with many skeins remaining coiled, resulting in an almost absent water retention. Therefore, at high ionic strength the presence of Ca^2+^ seems crucial to rupture all the vesicles within the deployment time frame, allowing to transmit mixing forces to the threads^27^ and thus form a functional slime network.

**Figure 6:**
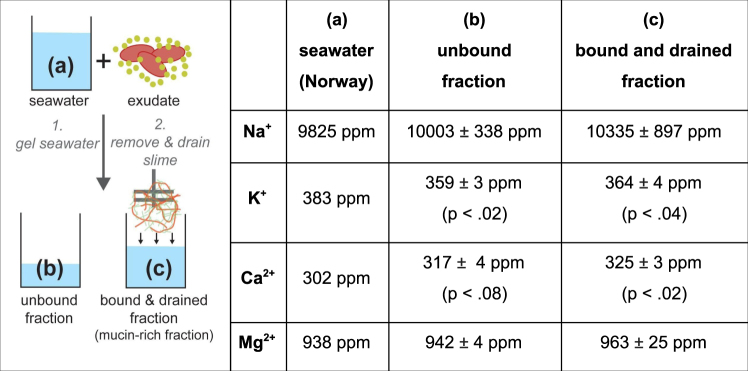
Dynamic cation concentrations during slime formation. The concentration of seawater cations (Na^+^, K^+^, Ca^2+^, Mg^2+^) of three seawater fractions - (**a**) seawater, (**b**) unbound seawater, and (**c**) bound and drained mucin-rich seawater - were analysed. The figure on the left represents a schematic drawing of the experiment. Concentrations are in parts per million (ppm).

The presence of 50 mM Mg^2+^ resulted in an only slightly inferior water retention to Ca^2+^ (Fig. 6b). Although it was found that Mg^2+^ only increased rupture in vesicles at about double the seawater concentration in seawater strength osmolarity^22^, it seems that for whole slime functionality Mg^2+^ has a similar effect to Ca^2+^. However, the origin of this beneficial effect is so far elusive. The similar water retentions between seawater and dilutions of seawater (Fig. 5a) imply that hagfish slime functionality is not limited to a narrow window of ion composition as long as specific divalent ions (Ca^2+^ and Mg^2+^) are present at concentrations similar to seawater. It was shown that >3 mM Ca^2+^ resulted in a significant increase in vesicle rupture^22^. Although in 1%/5% seawater there is only about 0.1/0.5 mM Ca^2+^, there might be a beneficiary effect of having additionally 0.5/2.5 mM Mg^2+^ present. Additionally, the low osmolarities of these dilutions could support a hypo-osmotic vesicle rupture and at the same time reduce thread swelling, allowing for controlled unraveling without tangling.

### Dynamic interactions of hagfish slime with seawater cations

Since hagfish slime deploys rapidly, it must distinctly interact with ions in the direct environment. To capture the dynamic processes between hagfish slime and seawater cations, the cation flux was investigated immediately after and five minutes after slime formation. Three fractions of liquids (a) seawater (Norway), the (b) unbound and the (c) bound & retained fraction were analyzed (Fig. 6). Hagfish slime significantly depleted potassium ions (K^+^) from seawater and released calcium ions (Ca^2+^). It was found that most K^+^ was depleted in the unbound fraction (−24 ppm, p < 0.02). Some K^+^ was again added to fraction (c) after five minutes of draining as fraction (c) showed an only 19 ppm (p < 0.04) lower concentration compared to seawater. Calcium followed an opposite trend as the unbound fraction showed some more calcium (+15 ppm, p < 0.08) whereas the bound and drained fraction showed significantly more calcium compared to seawater (+23 ppm, p < 0.02). Sodium and magnesium levels did not change significantly.

The depletion of K^+^ from seawater suggests that K^+^ is involved in an ion-exchange process during slime formation rather than for mucus gelation. The elevated Ca^2+^ levels in the fractions (b) and (c) (Fig. 6) raise the possibility that a K^+^/Ca^2+^ exchange process is involved in mucus decondensation during vesicle rupture, suggesting that the calcium is added by the ruptured vesicles. Skeins are unlikely to contribute substantial amounts of intracellular calcium when they unravel because cytoplasmic calcium levels are typically very low, and the skein develops within the cytoplasm of gland thread cells^4,5^. Apart from the skeins, Ca^2+^ can only be added by the vesicles as it is almost completely absent in the residual fluid^9^. A high intragranular calcium ion content of *M. glutinosa* mucus vesicles was suggested by Herr *et al*.^9^. The authors proposed that vesicle swelling is driven by a ‘jack-in-the-box’ mechanism, in which typically Ca^2+^-ions shield the charges of condensed polyanionic molecules such as mucin inside a vesicle^44^. This cation is Ca^2+^ in the case of mice mucin vesicles^45^ but can also be histamine for heparin or lysozyme for proteoglycans^44^. Once exposed to seawater, vesicle decondensation is triggered and Ca^2+^ is replaced by a less effective shielding cation such as Na^+^ or K^+^, causing repulsion between the negatively charged mucin polymers and thus fast swelling of the gel^46^. Our observations of dynamic cation concentrations in slime deployment support the suggestion of Herr *et al*.^9^ that hagfish mucin inside the vesicle is kept in a condensed state by Ca^2+^. Also, it is possible that Ca^2+^ is exchanged for K^+^ during vesicle swelling, as similarly reported by Nguyen *et al*.^47^ for mucus granules. The small potassium increase in fraction (c) compared to (b) supports the possible role of K^+^ as a counterion in mucin decondensation. The K^+^ ions do not seem to be strongly bound by the slime and drain again back into the solution. However, given the fact that sodium is present in seawater at 25× the concentration of potassium and therefore the diffusion of sodium would be faster, it seems unlikely that potassium exchange for calcium would evolve in preference to sodium. Therefore, probably both, potassium and sodium are exchanged for calcium during decondensation but the changes in sodium level could not be measured (see caveat further down) or the sulfonic groups of the mucin have a slight preference for potassium.

Furthermore, the fact that the mucin-rich fraction (c) in Fig. 6 contains higher calcium levels than fraction (b) suggests that Ca^2+^ binds to hagfish mucin and helps it to gel. The affinity of invertebrate mucus for calcium ions was shown before for mucus of the freshwater snail^48^. Ca^2+^-ions knowingly forms reversible cross-links and create salt-bridges between mucin chains, thus forming networks^49–51^. Therefore, the putative gelled mucin network interspaced in the thread network allows to entrap water. Furthermore, competitive binding of divalent cations over monovalent cations to sulfonated polyelectrolytes such as hagfish mucin^8^ is also well known^52–54^. The calcium probably bound to the mucin in a counterion condensation process^55–57^ and drained from the slime mass together with some of the mucin.

What is the amount of calcium that keeps the mucin condensed in the vesicle? 4 μl of exudate added about 0.3–0.46 mg calcium ions to the seawater. Calculating with an exudate density of about 1 mg/μl^10^ results in 75–115 μg Ca^2+^ per mg exudate. Considering that 66% of the exudate are residual fluid and 17% each are mucin vesicles or skeins^11^ about 0.68 mg mucins were added to the seawater. If all Ca^2+^ originates from the mucus vesicles 44 –68 wt% of the total mucus dry mass would be calcium. Calcium was shown to reach high intragranular levels between 2.5–3.6 moles calcium/kg dry mass mucus in the giant mucin granules of a slug (*Ariolimax columbianus*)^44^, corresponding to about 10–14.4 wt%. Considering that hagfish mucin must swell extremely fast in a defense situation against the high osmotic gradient of seawater, a roughly three to four times higher concentration than reported in slug mucin vesicles does not seem unlikely.

A caveat to the presented data lies in the high levels of sodium and magnesium in seawater, which limited an holistic insight in the dynamic ion flux during slime formation. Both calcium and potassium occur at concentrations of roughly 300–400 ppm whereas magnesium and sodium are present at more than double and ten-fold this concentration, respectively. We worked with concentrations close to the natural concentrations of hagfish exudate (1 mg exudate on 5 ml seawater^11^). Cation concentrations of 300–400 ppm result in a exudate/cation mass ratios of about 1/1.5–2 per cation as is the case for Ca^2+^ and K^+^. In contrast, this ratio is roughly 1/5 for Mg^2+^ and almost 1/50 for Na^+^. It is possible that the concentrations of Mg^2+^ and Na^+^ varied in the investigated fractions but their variation remained hidden in the small ratio of exudate/ion concentration. Future investigations such as measuring the calcium content only in the mucus and the skein fraction of the exudate or using dilutions of seawater and investigating the vesicles and skeins separately could help to provide a more detailed analysis of intragranular Ca^2+^ levels and ion flux during slime formation.

### Summary

**Figure 7:**
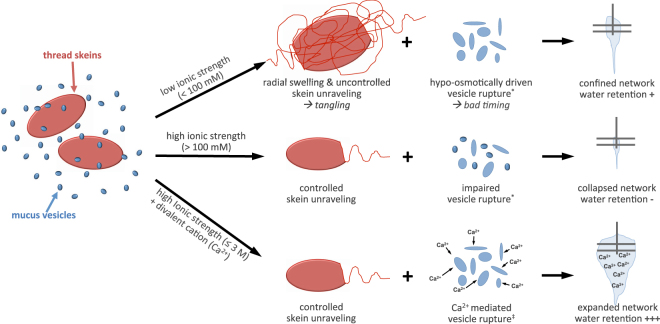
Schematic representation of the suggested role of ionic strength and divalent seawater cations (Ca^2+^) for the formation and functionality of hagfish slime. A low ionic strength causes the thread skeins to swell radially and unravel uncontrolled from both sides, causing tangling of the threads. The vesicles rupture due to the large osmotic gradient. Tangling combined with immediate vesicle rupture creates a confined thread network that fails to entrap large amounts of water. At a high ionic strength skein unraveling is controlled but vesicle rupture is impaired. A dense and collapsed network forms, resulting in an almost absent water retention. At a high ionic strength with Ca^2+^-ions present, skeins unravel controlled, vesicles rupture Ca^2+^-mediated, and the mucin probably gels. A widespread and expanded slime network forms that entraps large amounts of water as observed in seawater, resulting in a functional defensive hydrogel. Citations in the figure: *^9^; ^‡^^22^.

In this study we demonstrate the crucial role of ionic strength and seawater cations - especially Ca^2+^ - for the formation dynamics and functionality of hagfish slime. The findings are summarized and schematically depicted in Fig. 7. We suggest that sufficient ionic strength controls the dynamics of skein unraveling and slime network formation.

A low ionic strength caused a confined and narrow thread network in contrast to the widespread and expanded network formed in seawater. The thread skeins swelled and unraveled uncontrolled from both sides, probably causing tangling of the threads and thus preventing a widespread network. It is possible that the fast unraveling in ion-free water originates in an excessive swelling of the intermediate filament slime thread, which would possess increased stored strain energy. More stored strain energy would lead to a less controlled and faster unraveling. However, as the mucin vesicles ruptured in the hypo-osmotic environment of deionized water, a somewhat functional network that entraps about 50% of water in comparison to seawater can be formed in the absence of ionic strength.

At increased ionic strength (approx. >100 mM) a collapsed network formed that failed to incorporate water although the thread skeins unraveled controlled. We assume that as a consequence of impaired mucin vesicle rupture at high ionic strength - in the absence of calcium ions - an effective skein unraveling is limited as less mucus strands can attach to the threads to transmit mixing forces, leaving many skeins coiled. Only in the presence of divalent seawater cations Ca^2+^ and Mg^2+^ a functional slime network is realized at seawater strength osmolarity. Whereas the reasons for the beneficial effect of Mg^2+^ remain elusive, Ca^2+^ was shown to be important to mediate a complete and well-timed vesicle rupture, which supports skein unraveling in the high ionic strength environment, creating an expanded network. The presence of calcium allowed the formation of a functional slime network up to 3 M NaCl, corresponding to 4–5 times the ionic strength of seawater. Furthermore, Ca^2+^ could be necessary for an ionic gelation of hagfish mucin, which is supported by cation concentration measurements. These measurements further suggest that *M. glutinosa* mucin vesicles release intragranular Ca^2+^ during the rapid decondensation and swelling of hagfish mucin. Based on the findings in this work we propose that calcium has three distinct roles in hagfish slime: mucin condensation within vesicle, mucin decondensation via Ca^2+^-activated transporters in the vesicle membrane at high ionic strength^22^, and mucin gelation in the deployed slime.

Our results show that a functional defensive slime that entraps and retains water can only be formed in the presence of divalent seawater cations Ca^2+^ or Mg^2+^ at a high ionic strength. The insights on the interactions of hagfish slime with seawater ions will improve our understanding of the complex cascade of physico-chemical events underlying the formation of hagfish defensive slime and might support the design of bioinspired fibrous polyelectrolyte hydrogels that efficiently and rapidly form in high ionic strength environments.

## Electronic supplementary material

Movie S1 - Fishing Atlantic hagfish M. glutinosa in the Fjords of Alesund Norway

Movie S2 - Normal speed and slow-mo sequences of M. glutinosa skeins unraveling in Milli-Q

Movie S3 - Unraveling of M. glutinosa skeins in low ionic strength NaCl solutions and diluted seawater

Movie S4 - Unraveling of M. glutinosa skeins in high ionic strength solutions

Supplementary Information
