# Supplementary material for: Effect of ionic strength and seawater cations on hagfish slime formation
Source: Sci Rep. 2018 Jun 29;8:9867. doi: 10.1038/s41598-018-27975-0 (PMC6026207; doi:10.1038/s41598-018-27975-0)
Supplement: Supplementary file 5 — Supplementary Information [file 41598_2018_27975_MOESM5_ESM.pdf]

Supplementary Information

Effect of ionic strength and seawater cations on hagfish slime formation

L J Böni[1‡], R Zurflüh[1], M E Baumgartner[1], E J Windhab[1], P Fischer[1], S Kuster[1], and P A Rühs[2,3]

[1]Department of Health Science and Technology, ETH Zürich, 8092 Zürich, Switzerland

[2]Department of Materials, ETH Zürich, 8093 Zürich, Switzerland

[3]Department of Bioengineering and Materials Science and Engineering, University of California, Berkeley, California 94720-1760, USA

[‡]Corresponding author email: hagfish@ethz.ch

Supplementary Tables

Table S1: Recipe for artificial seawater (ASW) according to Kestner et al.[1].

Salts	Concentration (mM)
NaCl	409
Na_2SO_4	28
KCl	9
$MgCl_2$	53
$CaCl_2$	10
$NaHCO_3$	23

Table S2: Calculated ionic strength I for all used liquids. The concentration of the cations for the calculation of the ionic strength of the natural seawater from Norway was obtained in atomic absorption spectroscopy (AAS) measurements. The concentration of the anions (Cl^-, SO_4^{2-}, HCO_3^-) was estimated. For SO_4^{2-} and HCO_3^- the literature values of Kester et al.[1] were used. The concentration of Cl^- was determined using the concept of charge neutrality, i.e. the required remaining anions were considered chloride.

Liquid	I (mM)	I (M) rounded

Seawater from Norway	618	0.62
50% seawater from Norway	309	0.31
5% seawater from Norway	31	0.03
1% seawater from Norway	6	> 0.01
Artificial seawater (ASW)	696	0.70
ASW, no Na^+ and K^+	191	0.19
ASW, no Ca^{2+} and Mg^{2+}	505	0.51
ASW, no Ca^{2+}	665	0.67
ASW, no Mg^{2+}	536	0.54

Table S3: Comparison of the cationic composition of coastal seawater from Ålesund (Norway) with the recipe for artificial seawater (ASW) by Kestner et al.[1] in parts per million (ppm) and milli moles / liter (mM). One ppm equals 1 mg l^{-1}.

	coastal seawater (Ålesund, Norway)		artificial seawater		difference (%)
	ppm	mM	ppm	mM	
**Na^+**	9825	427.2	10766	468.1	+8.7%
**K^+**	383	9.8	356	9.1	-7.6%
**Ca^{2+}**	302	7.5	413	10.3	+26.8%
**Mg^{2+}**	938	38.6	1295	53.3	+27.6%

Supplementary Figures

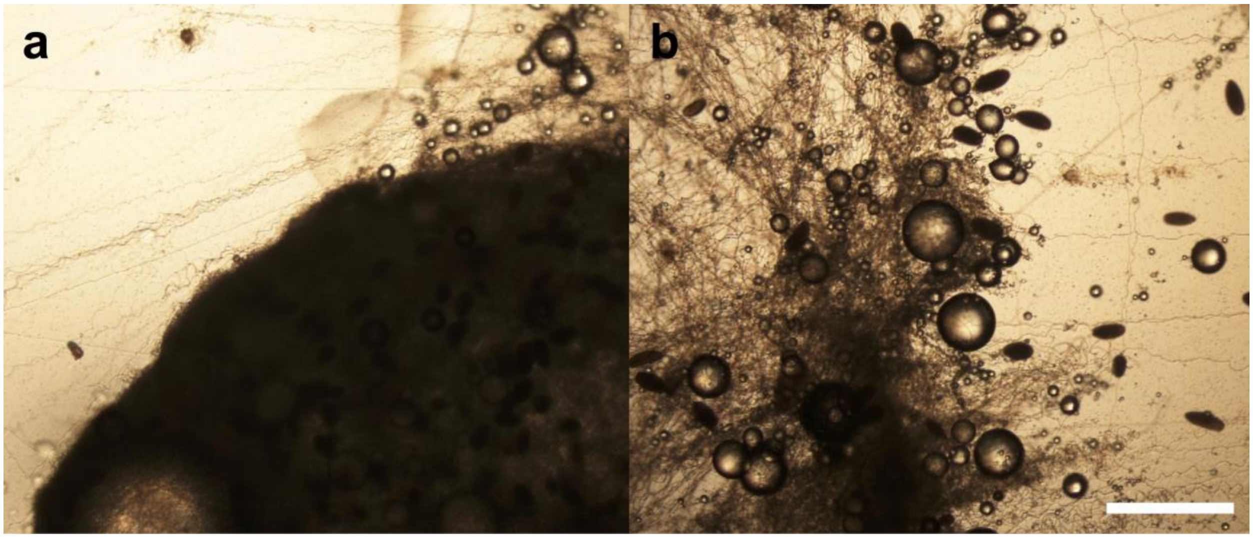

Figure S1: Light microscopy images of hagfish slime formed with 500 mM NaCl (**a**) and with artificial seawater without divalent cations (**b**) showing the dense and collapsed fiber network and the remaining coiled skeins. Scale bar = 500 μm.

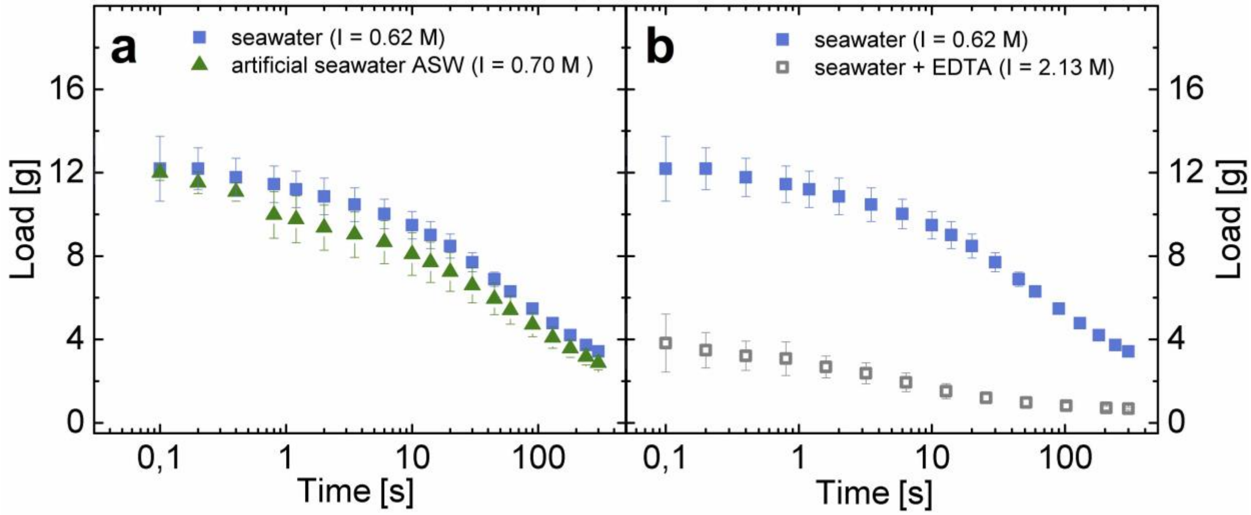

Figure S2: Water retention in seawater and artificial seawater (ASW) **(a)**. Water retention in seawater and in seawater with EDTA (150 mM) **(b)**. The pH of seawater with EDTA was adjusted to pH 8.0 using a 10 M NaOH solution in order to minimize the dilution.

Supplementary Movies:

Movie S1 - Fishing Atlantic hagfish (*M. glutinosa*) in the Fjords of Ålesund, Norway

Movie S2 - Normal speed and slow-mo sequences of *M. glutinosa* skeins unraveling in Milli-Q

Movie S3 - Unraveling of *M. glutinosa* skeins in low ionic strength NaCl solutions and diluted seawater

Movie S4 - Unraveling of *M. glutinosa* skeins in high ionic strength solutions

1. Kester, D. R., Duedall, I. W., Connors, D. N. & Pytkowicz, R. M. Preparation Of Artificial Seawater. *Limnol. Oceanogr.* **12,** 176–179 (1967).
